# Supplementary material for: NAD+ analog reveals PARP-1 substrate-blocking mechanism and allosteric communication from catalytic center to DNA-binding domains
Source: Nat Commun. 2018 Feb 27;9:844. doi: 10.1038/s41467-018-03234-8 (PMC5829251; doi:10.1038/s41467-018-03234-8)
Supplement: Supplementary file 1 — Supplementary Information [file 41467_2018_3234_MOESM1_ESM.pdf]

NAD$^+$ analog reveals PARP-1 substrate-blocking mechanism and allosteric communication from catalytic center to DNA-binding domains

Langelier et al.

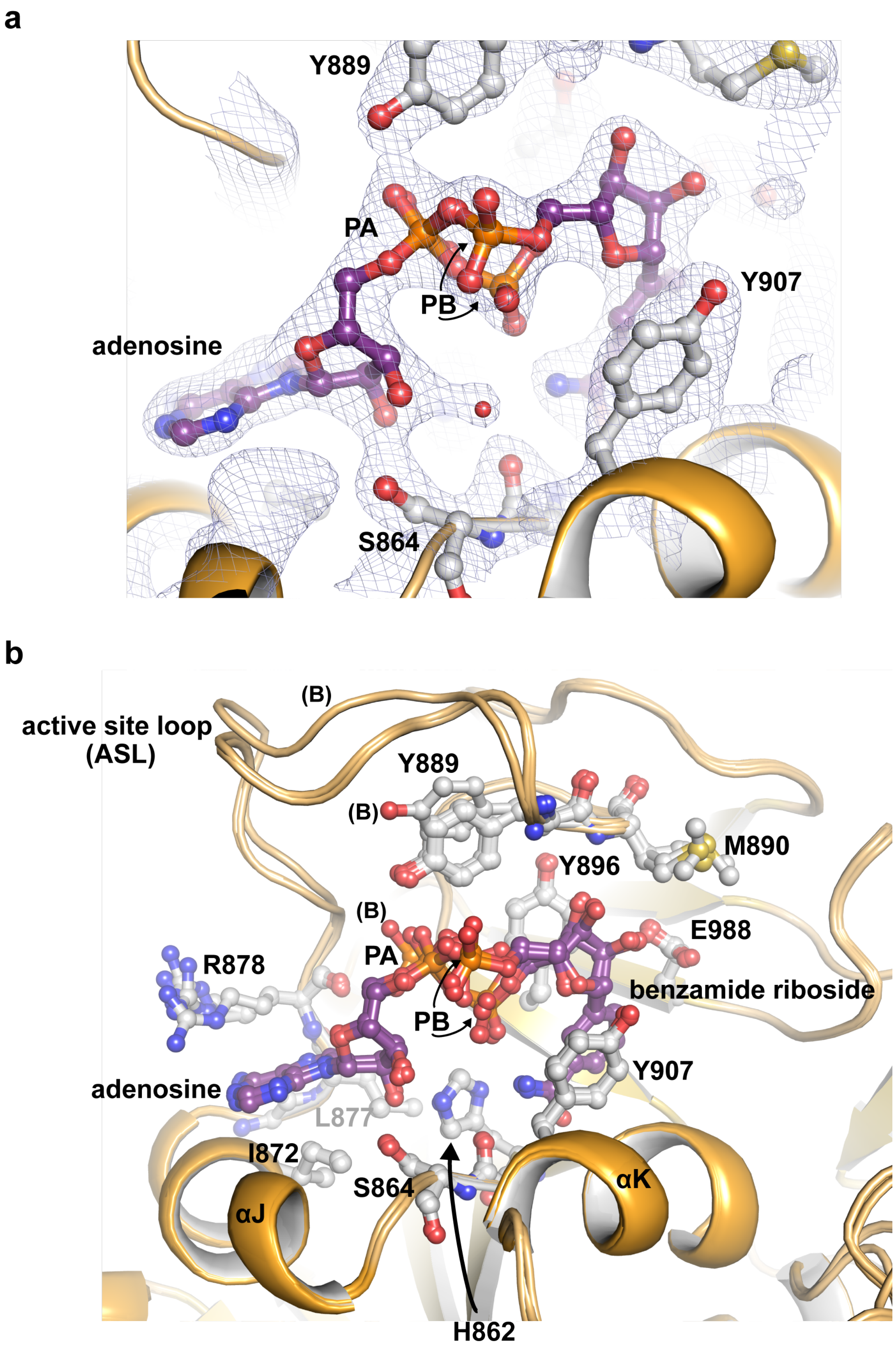
a
Y889
PA
PB
adenosine
Y907
S864
b
(B)
active site loop
(ASL)
Y889
(B)
M890
Y896
(B)
E988
PA
R878
benzamide riboside
PB
Y907
adenosine
L877
I872
S864
αK
αJ
H862

c

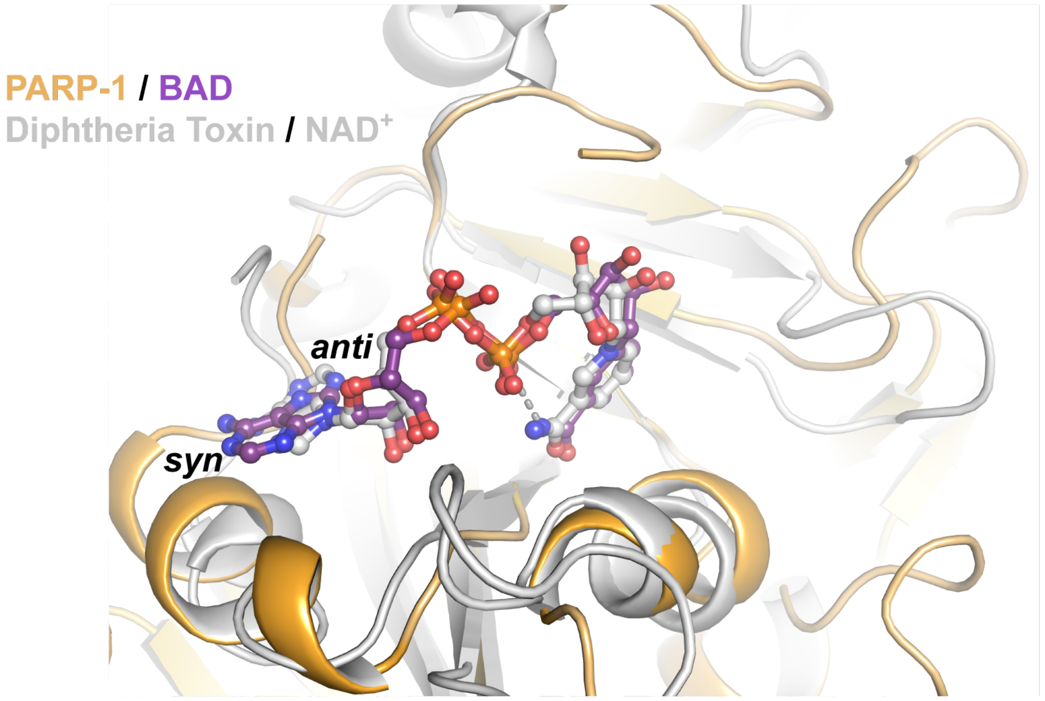
PARP-1 / BAD
Diphtheria Toxin / NAD+
anti
syn

d

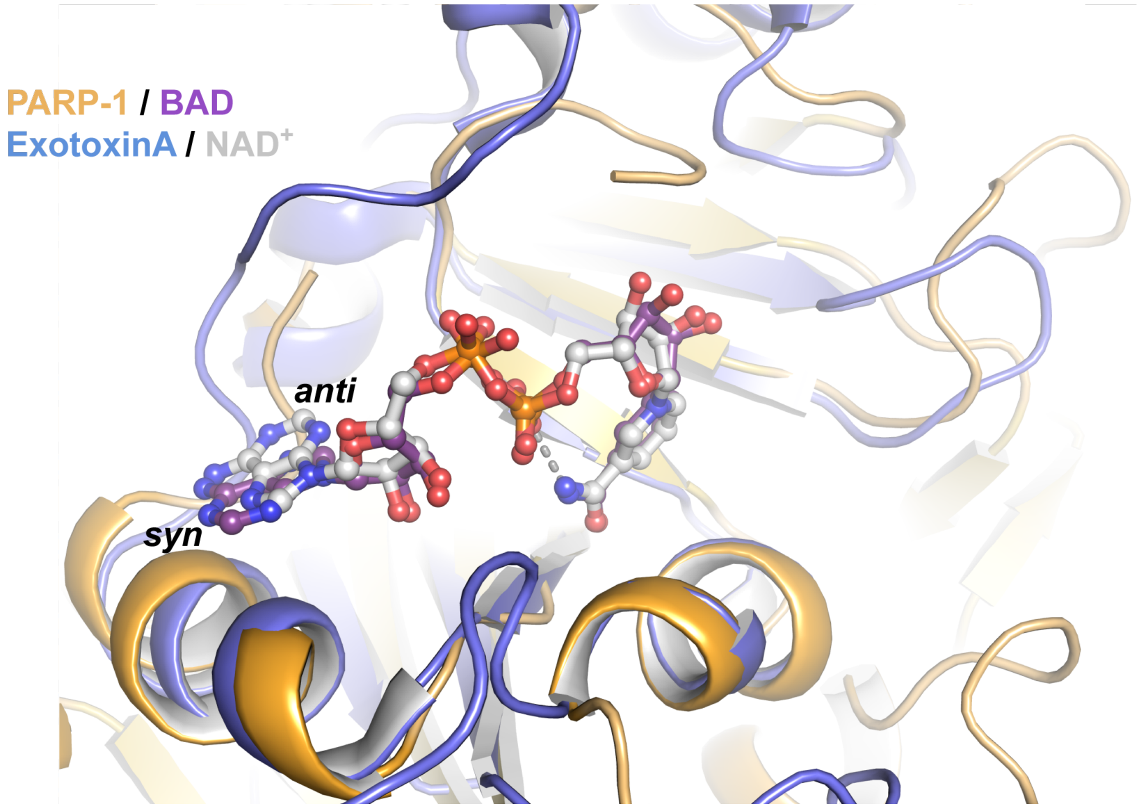
PARP-1 / BAD
ExotoxinA / NAD+
anti
syn

e

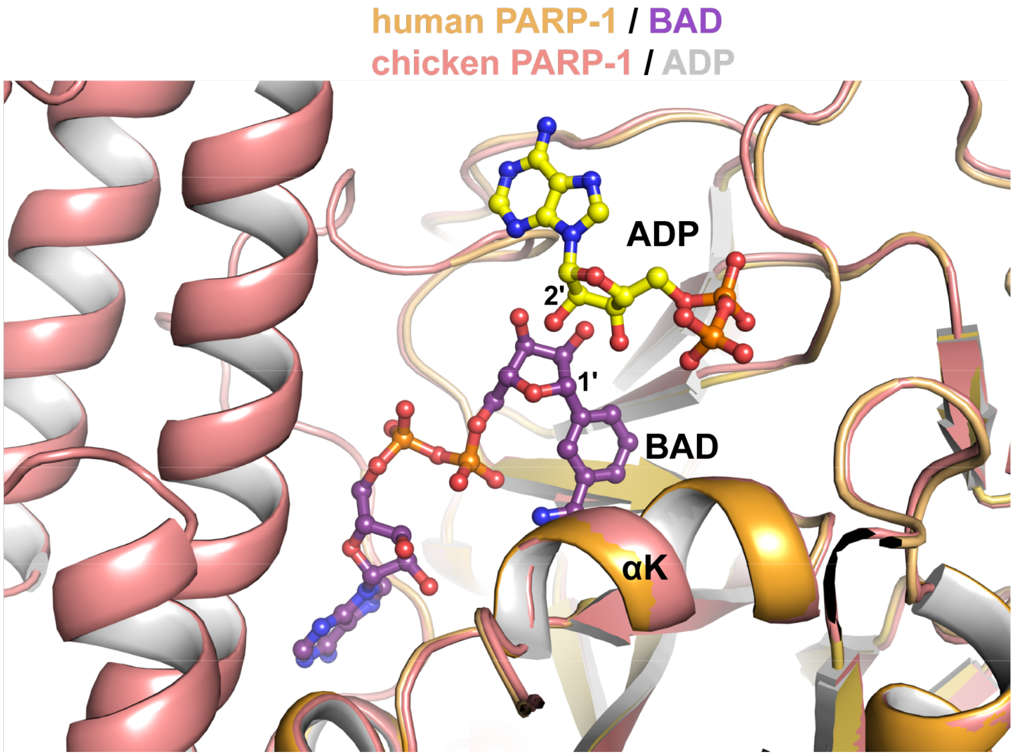

Supplementary Figure 1: Crystal structure of CAT ΔHD in complex with the non-hydrolyzable NAD$^+$ analog BAD.

a. 2.3 Å weighted electron density map contoured at 1.2σ in the region surrounding BAD bound to CAT ΔHD molecule D. Similar to molecules A and B, BAD bound to molecule D exhibited two conformations of the benzamide ribose phosphate (PB).

b. All four molecules of the crystal asymmetric unit (molecules A, B, C, D) are superimposed and drawn in cartoon mode with key amino acids drawn as ball and sticks and labeled. The benzamide riboside phosphates (PB) occupy two main conformations, whereas the adenosine phosphates (PA), the adenosine group, and the benzamide riboside occupy similar conformations. Molecules A, B, and D of PARP-1 each have a BAD molecule in the same single conformation observed for molecule C (see main Fig. 3a, b), but also have a second conformation in which PB is rotated away from H862 and the benzamide group. The second conformation of BAD in molecule B exhibits a slight translation in both PA and PB, and this appears to correlate with a repositioning of the active site loop and Y889. The conformations for molecule B are denoted by a B in parenthesis (B).

c, d. In panel **c**, the structure of Diphtheria toxin from *Corynebacterium diphtheriae* bound to NAD$^+$ (PDB code 1tox)[1] is superimposed on the structure of PARP-1 bound to BAD (molecule C). In panel d, the structure of Exotoxin A from *Pseudomonas aeruginosa* (PDB code 3b78)[2] is superimposed on the structure of PARP-1 bound to BAD (molecule C). Both alignments highlight that the BAD conformation represented in molecule C is most similar to the ADP-ribosylating toxins. The close proximity of the nicotinamide ribose phosphate of NAD$^+$ (PN; PB in BAD) to the nicotinamide ring creates a compact conformation that is proposed to aid in positioning of the nicotinamide ribose for reaction chemistry, and/or create a strain on the NAD$^+$ molecule that favors bond cleavage[3]. The adenine base in the Diptheria toxin and Exotoxin A structures is in the *anti* conformation, whereas the adenine base in the PARP-1/BAD structure is in the *syn* conformation.

e. The structure of chicken PARP-1 crystallized in the presence of carba-NAD$^+$ (PDB code 1a26)[4] is superimposed on the structure of PARP-1 bound to BAD. An ADP molecule was modeled in the structure, likely representing an ordered portion of the carba-NAD$^+$ molecule. The 2' hydroxyl of the ADP molecule is positioned above the benzamide ribose ring, near the 1' carbon of BAD, but also near the 2' and 3' hydroxyls. This approximate configuration has been proposed to support the elongation of ADP-ribose chains, which is composed of a 2' to 1' ribose-ribose linkage. A more complete understanding of the reaction chemistry of protein ADP-ribosylation and ADP-ribose chain formation will require ternary complexes of PARP-1, NAD$^+$, and a peptide presenting an acceptor amino acid or a poly-ADP-ribose chain as an acceptor, respectively.

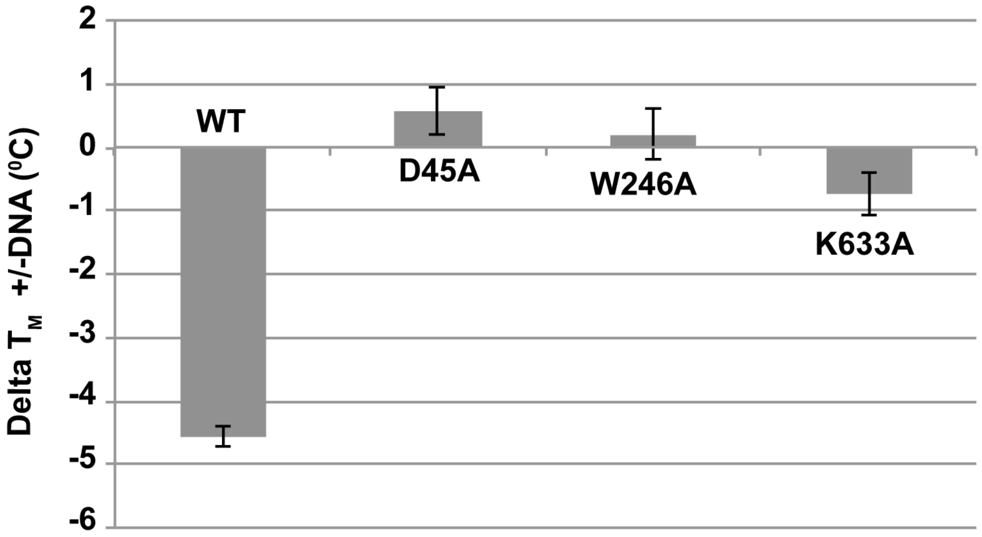

Supplementary Figure 2: DSF experiment to monitor HD destabilization/unfolding in response to DNA binding.

The T_M of full-length PARP-1 WT and interdomain mutants (5 μM) were measured in the absence and presence of DNA (2.5 μM) by DSF. The delta T_M was obtained by subtracting the T_M measured in the absence of DNA from the T_M measured in the presence of DNA. The values shown are the average of three independent experiments with the associated standard deviation.

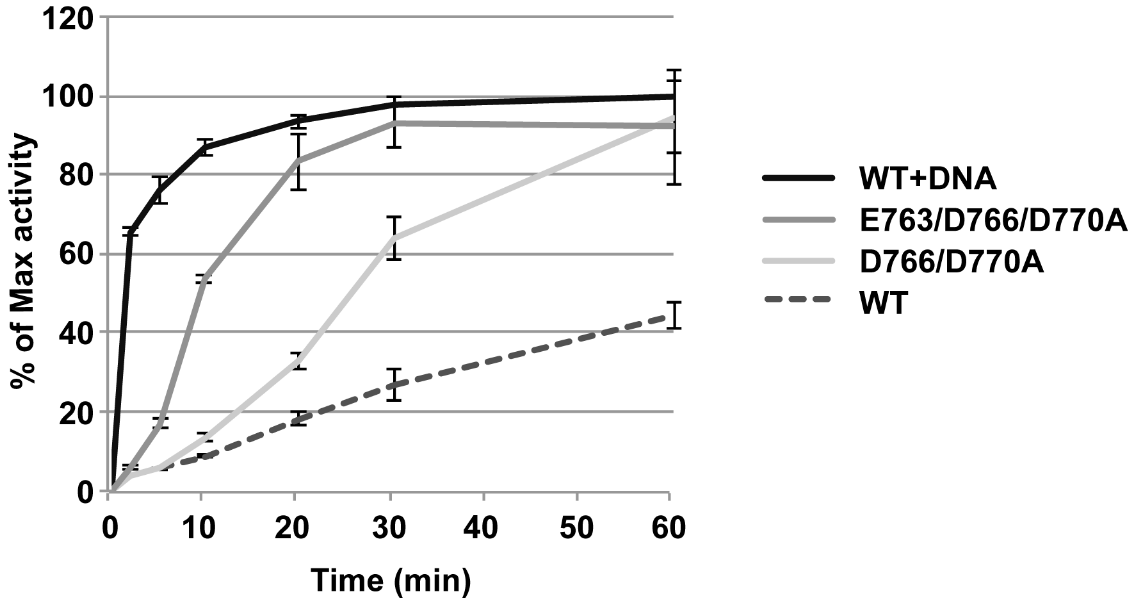

Supplementary Figure 3: Colorimetric activity assay for PARP-1 WT, D766/D770A, and E763/D766/D770A.

The activity of full-length PARP-1 WT and mutants (20 nM) was measured using the colorimetric assay. The activity of PARP-1 WT in the presence of DNA (40 nM) is shown to compare with the level of activity of the overactive mutants in the absence of DNA. The values are an average of three independent experiments with the associated standard deviation.

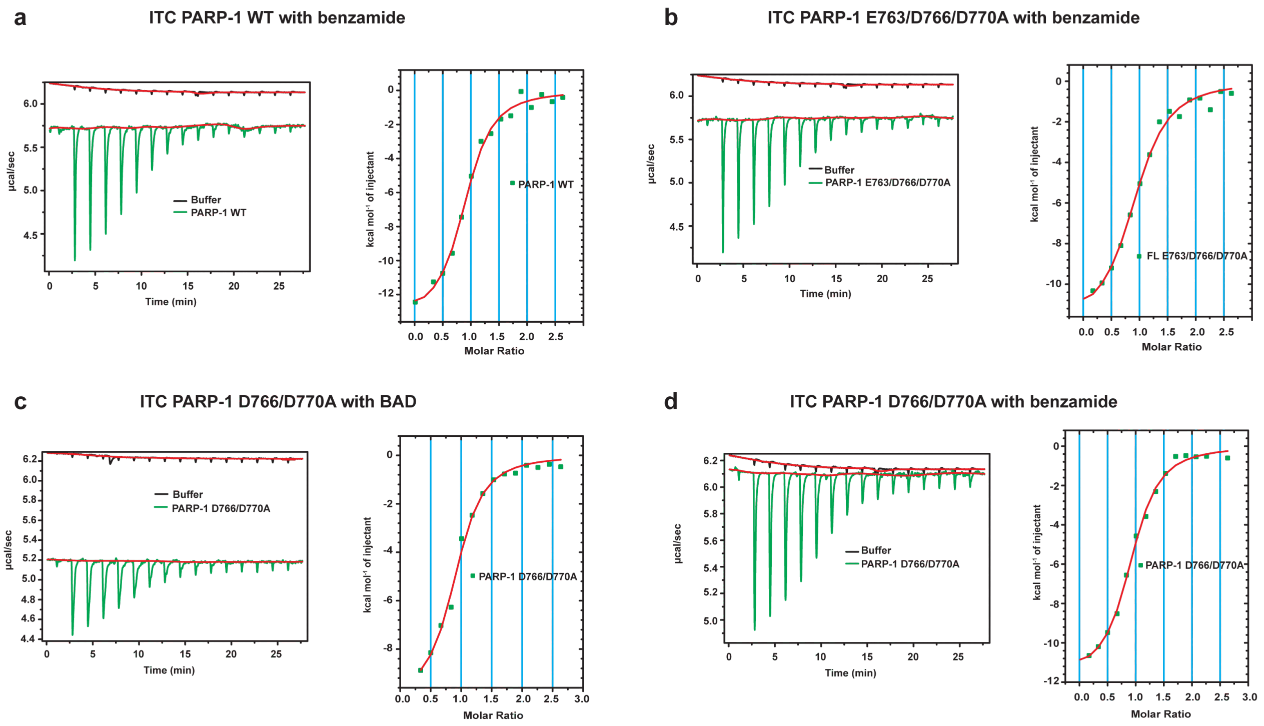

Supplementary Figure 4: ITC experiments for full-length PARP-1 WT and mutants.

a. ITC results where benzamide (525 µM in the syringe) was titrated into PARP-1 WT (40 µM in the cell).

b. Same conditions as in panel **a** using PARP-1 E763/D766/D770A.

c. ITC results where BAD (525 µM in the syringe) was titrated into PARP-1 D766/D770A (40 µM).

d. Same conditions as in panel **c** using benzamide.

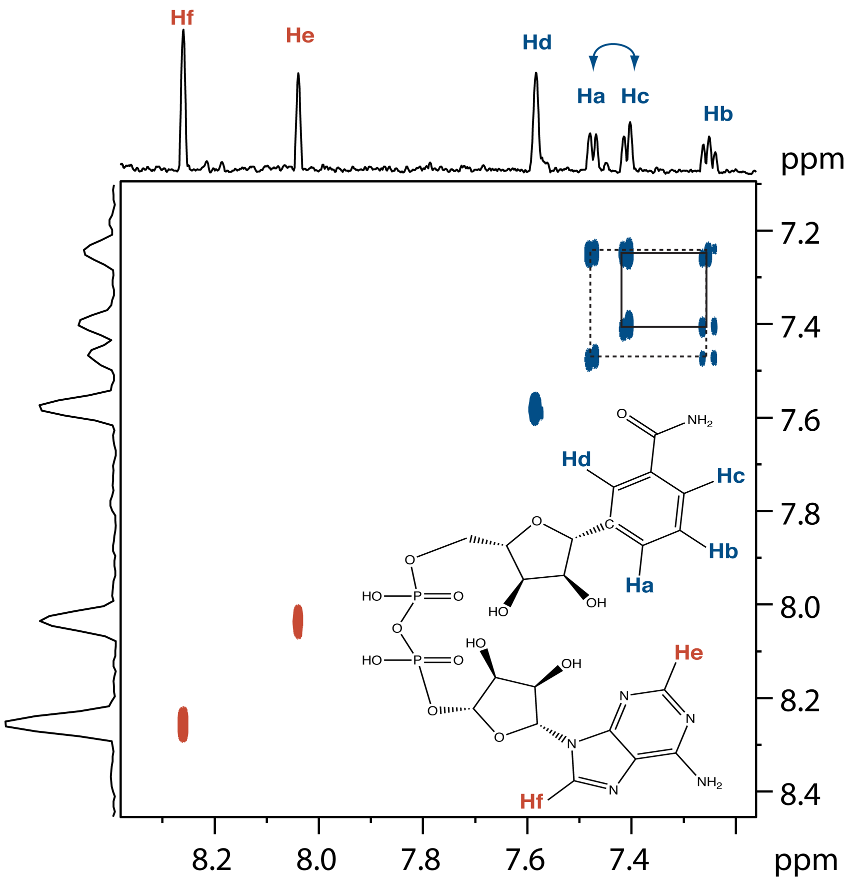

Supplementary Figure 5: 2D ^{1}H-^{1}H correlation (COSY) spectrum along with the chemical structure of BAD.

Peaks assignments are made on the basis of correlations and comparison of observed chemical shifts with those reported for NAD^{+} (Biological Magnetic Resonance Data Bank entry: BMSE000053). Some ambiguity exists in assignment of Ha and Hc.

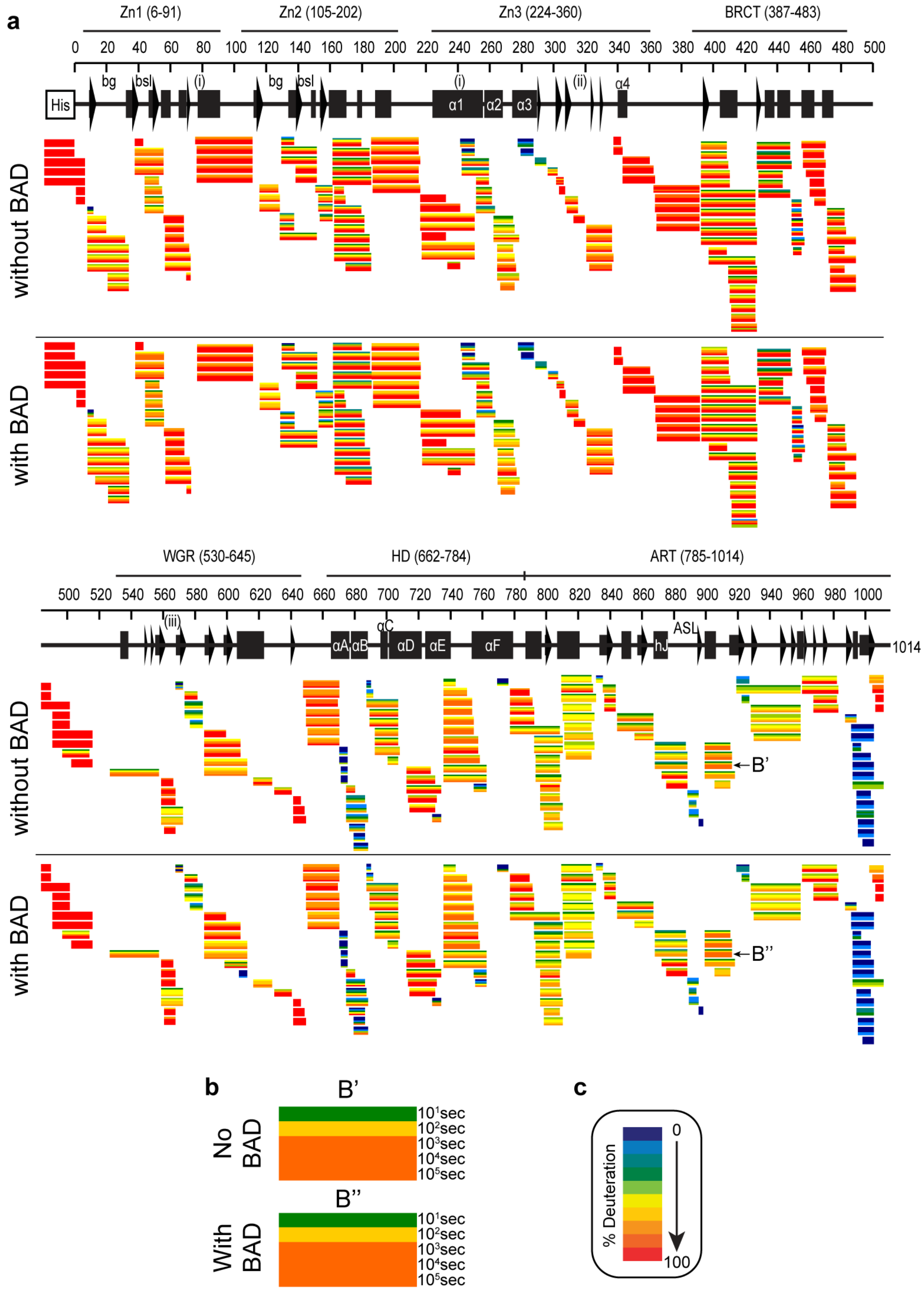
a
Zn1 (6-91)
Zn2 (105-202)
Zn3 (224-360)
BRCT (387-483)
His
bg
bsl
(i)
(ii)
α1
α2
α3
α4
without BAD
with BAD
WGR (530-645)
HD (662-784)
ART (785-1014)
(iii)
αA
αB
αC
αD
αE
αF
hJ
ASL
1014
B'
B''
b
No BAD
With BAD
10^1sec
10^2sec
10^3sec
10^4sec
10^5sec
c
% Deuteration
0
100

Supplementary Figure 6. Effect of BAD on PARP-1 dynamics in the absence of DNA measured by HXMS.

a. HXMS data for PARP-1 with or without BAD in the absence of DNA. Each horizontal bar represents an individual peptide, and the 5 stripes within each bar are colored according to percentage deuteration at each of the 5 time points (10^1 s, 10^2 s, 10^3 s, 10^4 s, 10^5 s). bg, backbone grip; bsl, base stacking loop; (i) F1-F3 interface; (ii) Z3/WGR/HD interface; (iii) WGR/HD interface.

b. The peptides indicated in panel **a** as B' and B" are displayed in the absence of BAD (top) and in the presence of BAD (bottom), respectively.

c. Color scheme for panels **a** and **b**.

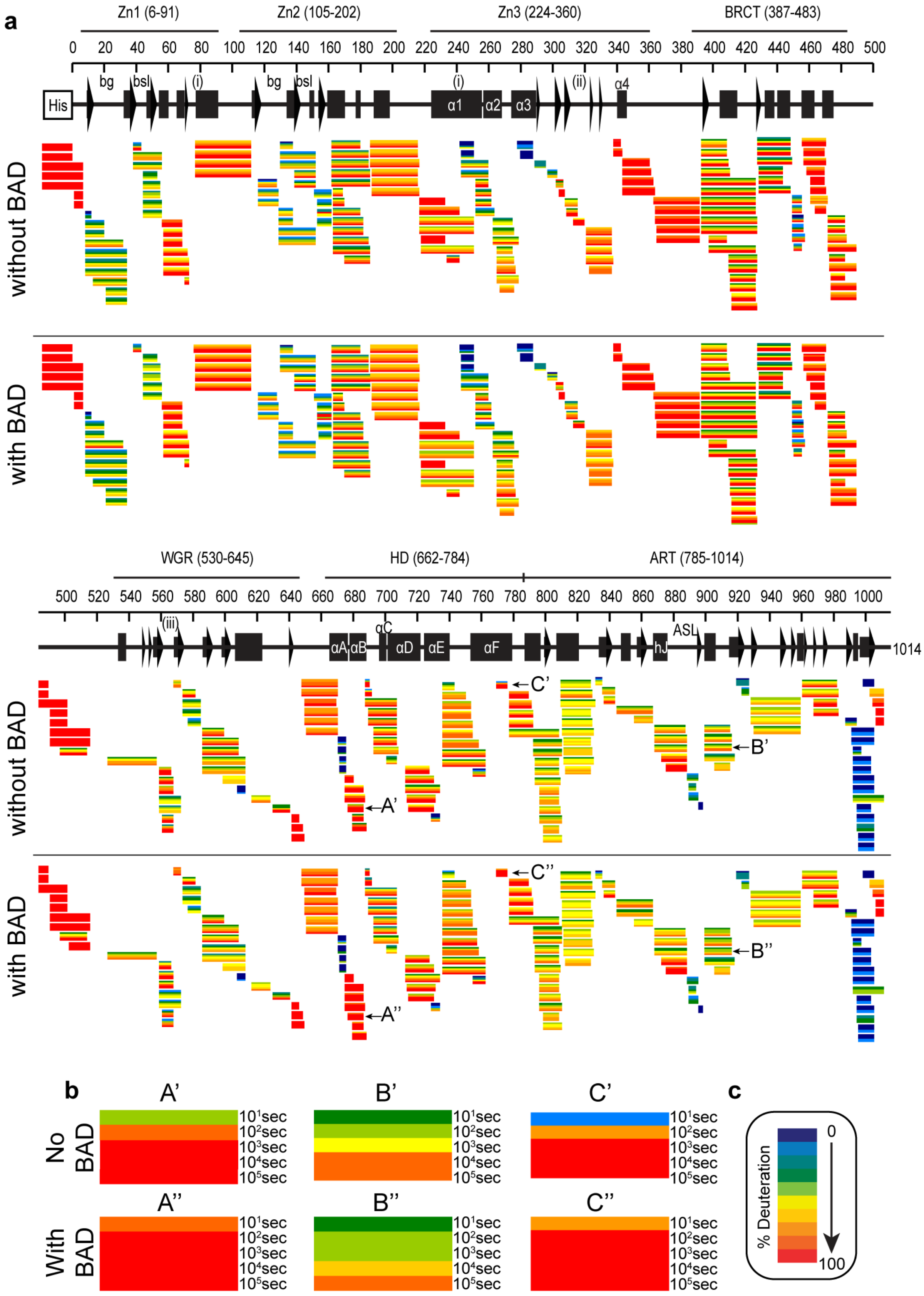
a
Zn1 (6-91)
Zn2 (105-202)
Zn3 (224-360)
BRCT (387-483)
bg
bsl
(i)
(ii)
α1
α2
α3
α4
His
without BAD
with BAD
WGR (530-645)
HD (662-784)
ART (785-1014)
(iii)
αA
αB
αC
αD
αE
αF
hJ
ASL
1014
C'
C''
A'
A''
B'
B''
b
No BAD
With BAD
10¹sec
10²sec
10³sec
10⁴sec
10⁵sec
c
% Deuteration
0
100

Supplementary Figure 7. Effect of BAD on PARP-1 dynamics in the presence of DNA measured by HXMS.

a. HXMS data for PARP-1 with or without BAD in the presence of DNA. Each horizontal bar represents an individual peptide, and the 5 stripes within each bar are colored according to percentage deuteration at each of the 5 time points (10^1 s, 10^2 s, 10^3 s, 10^4 s, 10^5 s). bg, backbone grip; bsl, base stacking loop; (i) F1-F3 interface; (ii) Z3/WGR/HD interface; (iii) WGR/HD interface.

b. The peptides indicated in panel **a** (i.e. A', A", B', B", C', and C") are displayed in the absence of BAD (top; A', B', C') and in the presence of BAD (bottom; A", B", C").

c. Color scheme for panels **a** and **b**.

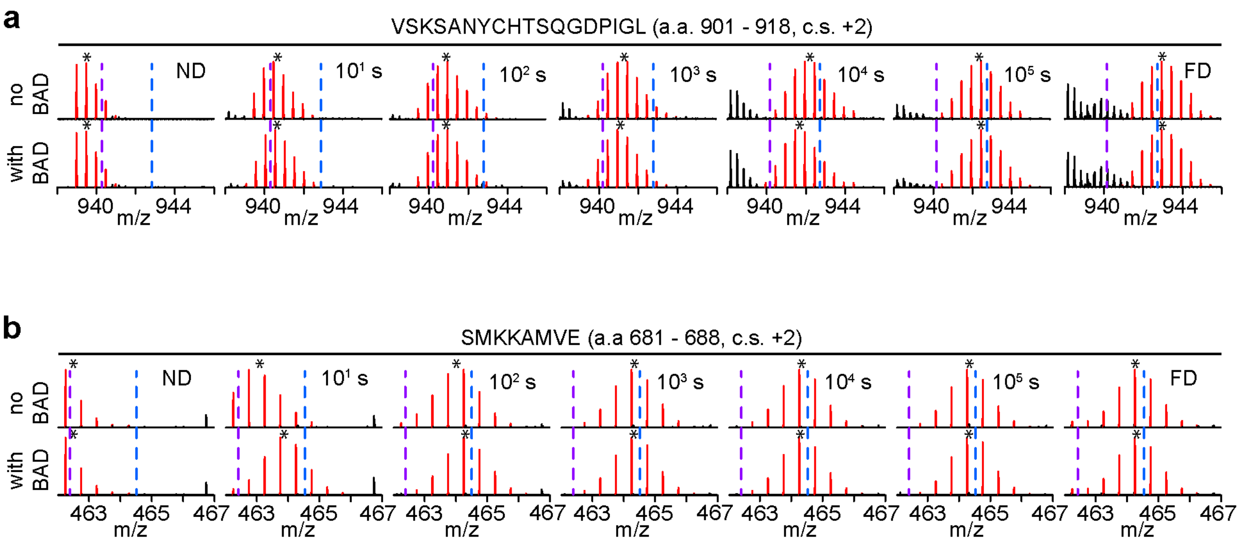

Supplementary Figure 8. Raw mass spectra of representative peptides from the NAD^+ binding site (a) and from the αB of the HD domain (b), all in the presence of DNA and in the presence or absence of BAD.

Red isotopic envelopes represent the indicated peptides, while black isotopic envelopes are from other co-eluting peptides (note their charge states differ from the peptides of interest) in the same m/z region. Centroid values are indicated using asterisks. Blue and purple dotted lines serve as guides for visualizing differences. ND represents the non-deuterated sample. FD represents the "fully-deuterated" sample. The protection from HX for the peptide from the CAT domain can be most strongly observed in panel **a** at 10^4 sec where the isotopic envelope is less deuterated in the presence of BAD. HD unfolding due to BAD binding is clear in panel **b**, where the peptide is almost fully deuterated in the presence of BAD even after just 10^1 sec.

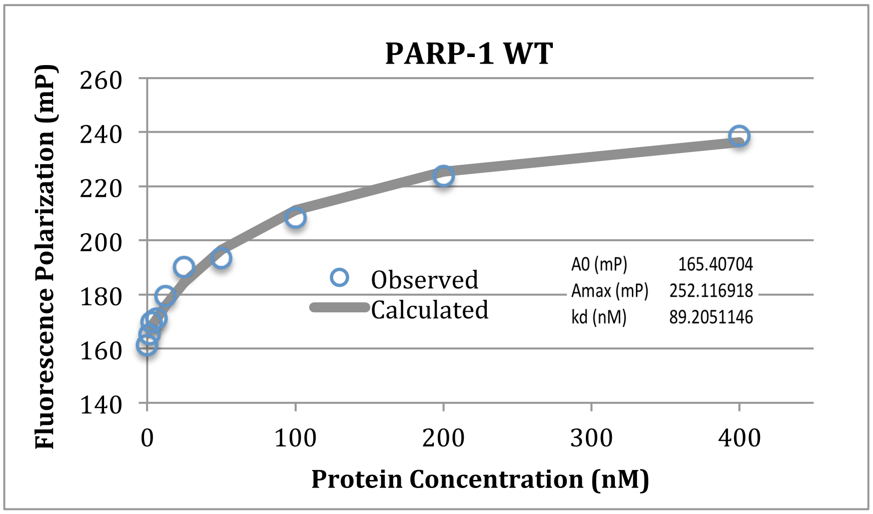
PARP-1 WT
Fluorescence Polarization (mP)
260
240
220
200
180
160
140
0
100
200
300
400
Protein Concentration (nM)
Observed
Calculated
A0 (mP) 165.40704
Amax (mP) 252.116918
kd (nM) 89.2051146

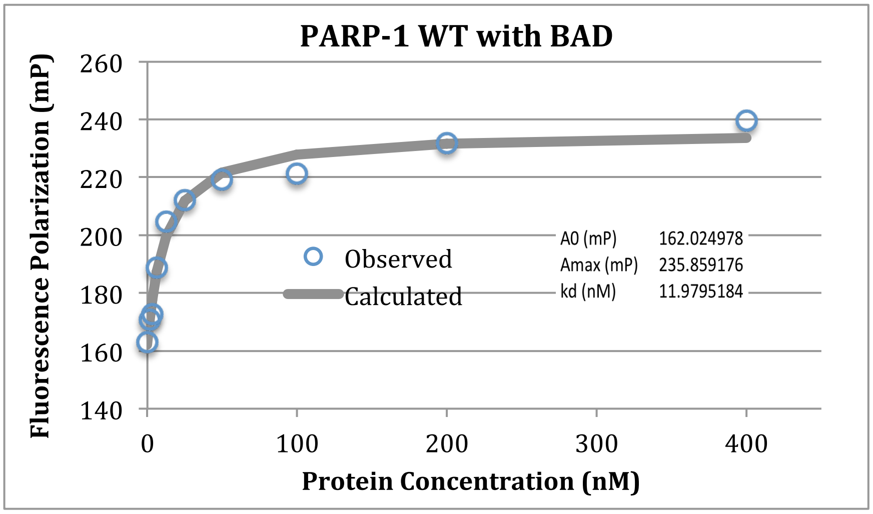
PARP-1 WT with BAD
Fluorescence Polarization (mP)
260
240
220
200
180
160
140
0
100
200
300
400
Protein Concentration (nM)
Observed
Calculated
A0 (mP) 162.024978
Amax (mP) 235.859176
kd (nM) 11.9795184

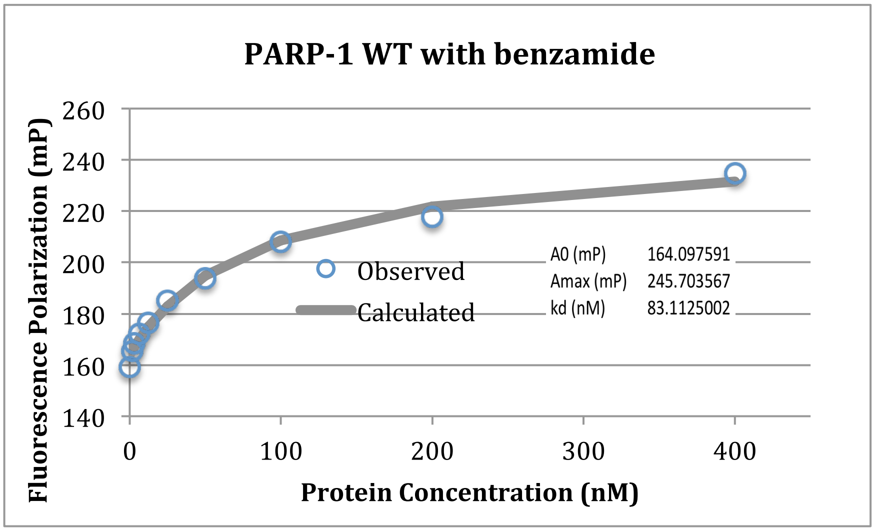
PARP-1 WT with benzamide
Fluorescence Polarization (mP)
260
240
220
200
180
160
140
0
100
200
300
400
Protein Concentration (nM)
Observed
Calculated
A0 (mP) 164.097591
Amax (mP) 245.703567
kd (nM) 83.1125002

Supplementary Figure 9. PARP-1 affinity for DNA is increased in the presence of BAD. A fluorescence polarization DNA binding assay was used to measure the affinity of PARP-1 for a DNA dumbbell probe containing a single nucleotide central nick in the absence of compound or in the presence of BAD or benzamide. A representative graph of the fitted data is shown for each experiment. Observed data measurements are shown with open spheres, and the fitting of a two-state binding model to the data is shown with a solid grey line. Fitted parameters: plateau of maximum polarization, Amax; baseline polarization of probe, A0; apparent equilibrium binding constant, kd.

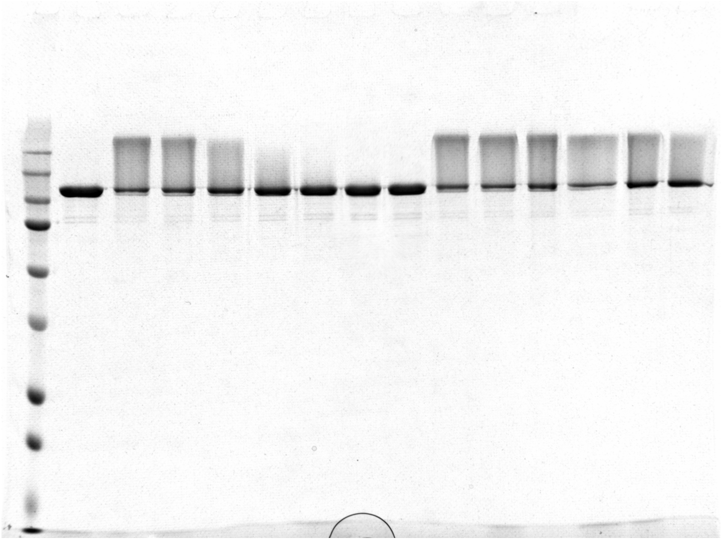

Supplementary Figure 10. Uncropped version of the gel presented in Figure 1c.
A Precision Plus Protein Standard (BioRad) is loaded in the first lane of the gel (left). The visible bands of the marker are as follows, starting at the top of the gel lane:
250 kDa, 150 kDa, 100 kDa, 75 kDa, 50 kDa, 37 kDa, 25 kDa, 20 kDa, and 15 kDa.

Supplementary Table 1

**Figure 1d**	T_M	+/–
CAT WT	51.23	0.18
CAT WT+ BAD 50 µM	51.06	0.19
CAT WT+ BAD 250 µM	51.09	0.13
CAT WT+ BAD 1250 µM	51.30	0.22
CAT WT+ carba-NAD+ 50 µM	51.29	0.29
CAT WT+ carba-NAD+ 250 µM	51.17	0.15
CAT WT+ carba-NAD+ 1250 µM	51.28	0.10
CAT ΔHD	41.76	0.34
CAT ΔHD+ BAD 50 µM	47.02	0.06
CAT ΔHD+ BAD 250 µM	49.58	0.10
CAT ΔHD+ BAD 1250 µM	51.91	0.12
CAT ΔHD+ carba-NAD+ 50 µM	41.75	0.23
CAT ΔHD+ carba-NAD+ 250 µM	41.83	0.24
CAT ΔHD+ carba-NAD+ 1250 µM	42.40	0.21
**Figure 1e**	T_M	+/–
CAT WT	51.58	0.23
CAT WT+benzamide 25 µM	52.53	0.15
CAT WT+benzamide 150 µM	54.28	0.16
CAT WT+benzamide 750 µM	54.86	0.16
CAT WT+benzamide 1500 µM	57.12	0.20
CAT WT+ADP-ribose 25 µM	51.52	0.09
CAT WT+ADP-ribose 150 µM	51.61	0.18
CAT WT+ADP-ribose 750 µM	51.56	0.16
CAT WT+ADP-ribose 1500 µM	51.53	0.26
CAT WT+BAD 25 µM	51.48	0.15
CAT WT+BAD 150 µM	51.45	0.08
CAT WT+BAD 750 µM	51.64	0.18
CAT WT+BAD 1500 µM	51.60	0.24
CAT ΔHD	41.26	0.12
CAT ΔHD+benzamide 25 µM	43.73	0.47
CAT ΔHD+benzamide 150 µM	46.20	0.46
CAT ΔHD+benzamide 750 µM	46.99	0.44
CAT ΔHD+benzamide 1500 µM	49.98	0.44
CAT ΔHD+ADP-ribose 25 µM	41.46	0.52
CAT ΔHD+ADP-ribose 150 µM	41.57	0.47
CAT ΔHD+ADP-ribose 750 µM	41.43	0.60
CAT ΔHD+ADP-ribose 1500 µM	41.68	0.23
CAT ΔHD+BAD 25 µM	45.52	0.48
CAT ΔHD+BAD 150 µM	48.60	0.56
CAT ΔHD+BAD 750 µM	49.27	0.54
CAT ΔHD+BAD 1500 µM	51.95	0.47

**Figure 3a**	T_M	+/–
WT	48.17	0.04
WT BAD	48.19	0.24
WT DNA	44.35	0.29
WT DNA BAD	47.65	0.56
D766/D770A	45.94	0.47
D766/D770A BAD	48.50	0.48
E763/D766/D770A	45.74	0.48
E763/D766/D770A BAD	50.13	0.15
**Figure 3b and Supp. Figure 2**	T_M	+/–
WT	47.41	0.32
WT BAD	47.09	0.43
WT DNA	42.85	0.21
WT DNA BAD	45.99	0.26
D45A	46.20	0.41
D45A BAD	46.68	0.47
D45A DNA	46.77	0.05
D45A DNA BAD	46.61	0.21
W246A	47.24	0.11
W246A BAD	47.23	0.23
W246A DNA	47.45	0.46
W246A DNA BAD	47.41	0.15
K633A	45.50	0.94
K633A BAD	45.32	1.06
K633A DNA	44.76	1.06
K633A DNA BAD	44.86	1.12
**Figure 3c**	T_M	+/–
PARP-1	47.93	0.18
PARP-1 BAD	48.15	0.34
PARP-1 DNA	44.45	0.08
PARP-1 DNA BAD	48.03	0.30
PARP-2	47.71	0.16
PARP-2 BAD	47.47	0.17
PARP-2 DNA	45.44	0.24
PARP-2 DNA BAD	47.28	0.07
PARP-3	35.81	0.12
PARP-3 BAD	35.54	0.20
PARP-3 DNA	39.70	0.33
PARP-3 DNA BAD	41.02	0.23
**Figure 3d**	T_M	+/–
PARP-1 CAT	51.04	0.17
PARP-1 CAT BAD	51.21	0.22
TNK CAT	53.71	0.02
TNK CAT BAD	57.42	0.02

Supplementary Table 1: Melting temperature (T_M) of PARP-1 WT and mutants measured by DSF. DSF was used to measure the T_M of PARP-1 WT and mutants alone or in the presence of the indicated compounds and/or DNA. The T_M values are an average of 3 independent experiments with the associated standard deviation (+/–). The data is arranged with regards to the associated figure in the main text, as indicated.

Supplementary References

1. Bell, C. E. & Eisenberg, D. Crystal Structure of Diphtheria Toxin Bound to Nicotinamide Adenine Dinucleotide †. *Biochemistry* **35,** 1137–1149 (1996).
2. Jørgensen, R., Wang, Y., Visschedyk, D. & Merrill, A. R. The nature and character of the transition state for the ADP-ribosyltransferase reaction. *EMBO Rep.* **9,** 802–9 (2008).
3. Bell, C. E., Yeates, T. O. & Eisenberg, D. Unusual conformation of nicotinamide adenine dinucleotide (NAD) bound to diphtheria toxin: a comparison with NAD bound to the oxidoreductase enzymes. *Protein Sci.* **6,** 2084–2096 (1997).
4. Ruf, a, Rolli, V., de Murcia, G. & Schulz, G. E. The mechanism of the elongation and branching reaction of poly(ADP-ribose) polymerase as derived from crystal structures and mutagenesis. *J. Mol. Biol.* **278,** 57–65 (1998).
